# MRI plaque imaging reveals high-risk carotid plaques especially in diabetic patients irrespective of the degree of stenosis

**DOI:** 10.1186/1471-2342-10-27

**Published:** 2010-11-30

**Authors:** L Esposito, T Saam, P Heider, Angelina Bockelbrink, Jaroslav Pelisek, D Sepp, R Feurer, C Winkler, T Liebig, K Holzer, O Pauly, S Sadikovic, B Hemmer, H Poppert

**Affiliations:** 1 Department of Neurology, Klinikum rechts der Isar, Technische Universität, Munich, Germany; 2 Department of Radiology, Ludwig Maximilians Universität, Munich, Germany; 3 Department of Vascular Surgery, Klinikum rechts der Isar, Technische Universität, Munich, Germany; 4 Social Medicine, Epidemiology, and Health Economics, Charité University, Berlin, Germany; 5 Department of Neuroradiology, Klinikum rechts der Isar, Technische Universität, Munich, Germany; 6 Department of Informatics, Klinikum rechts der Isar, Technische Universität, Munich, Germany

## Abstract

**Background:**

Plaque imaging based on magnetic resonance imaging (MRI) represents a new modality for risk assessment in atherosclerosis. It allows classification of carotid plaques in high-risk and low-risk lesion types (I-VIII). Type 2 diabetes mellitus (DM 2) represents a known risk factor for atherosclerosis, but its specific influence on plaque vulnerability is not fully understood. This study investigates whether MRI-plaque imaging can reveal differences in carotid plaque features of diabetic patients compared to nondiabetics.

**Methods:**

191 patients with moderate to high-grade carotid artery stenosis were enrolled after written informed consent was obtained. Each patient underwent MRI-plaque imaging using a 1.5-T scanner with phased-array carotid coils. The carotid plaques were classified as lesion types I-VIII according to the MRI-modified AHA criteria. For 36 patients histology data was available.

**Results:**

Eleven patients were excluded because of insufficient MR-image quality. DM 2 was diagnosed in 51 patients (28.3%). Concordance between histology and MRI-classification was 91.7% (33/36) and showed a Cohen's kappa value of 0.81 with a 95% CI of 0.98-1.15. MRI-defined high-risk lesion types were overrepresented in diabetic patients (n = 29; 56.8%). Multiple logistic regression analysis revealed association between DM 2 and MRI-defined high-risk lesion types (OR 2.59; 95% CI [1.15-5.81]), independent of the degree of stenosis.

**Conclusion:**

DM 2 seems to represent a predictor for the development of vulnerable carotid plaques irrespective of the degree of stenosis and other risk factors. MRI-plaque imaging represents a new tool for risk stratification of diabetic patients.

See Commentary: http://www.biomedcentral.com/1741-7015/8/78/abstract

## Background

MRI-based plaque imaging represents a new noninvasive imaging technique to reliably analyze plaque features of patients presenting with carotid artery stenosis[1-3]. Based on histological American Heart Association (AHA) criteria, modified specifically for MRI use, a classification was introduced by Cai and co-workers[4] that allows categorization of carotid plaques noninvasively into distinct lesion types (I-VIII). According to this modified classification, plaque lesions characterized by a lipid-rich necrotic core, by the presence of a thinned fibrous cap or by intraplaque hemorrhage represent lesion types IV-VI and are regarded as high-risk, unstable plaques that are likely to rupture and lead to cerebral ischemia[5-15]. In a previous study we showed that these high-risk lesion types IV-VI were indeed more prevalent in patients presenting with symptomatic carotid artery stenosis compared with asymptomatic patients[16].

The vulnerability of atherosclerotic plaque might be influenced by risk factors promoting the development of atherosclerotic changes, and diabetes mellitus (DM) in particular is known to be associated with a specific pattern of vascular change[17]. Although DM represents a well-established risk factor for atherosclerosis[18,19], its specific influence on plaque vulnerability in carotid artery stenosis is not clear. Previous reports suggest that atherosclerosis due to diabetes is different from the atherosclerotic pattern caused by other risk factors [20-23]. Since atherosclerotic features like vasa vasorum neovascularisation, intraplaque hemorrhage and lipid-core expansion seem to represent specific diabetic plaque features[18], diabetes might play an important role in plaque remodeling and in the development of plaque vulnerability. However, studies analyzing carotid plaque morphology based on MRI plaque imaging in relation to type 2 diabetes (DM 2) are rare.

It is the aim of this study to investigate whether MRI-based plaque imaging has the ability to detect possible differences in carotid plaque features of diabetic patients compared with nondiabetic patients. In addition, we wanted to analyze whether diabetic patients with MRI-detected high-risk lesion types are at higher risk than nondiabetic patients with high risk lesion types for the development of cerebral ischemia after endarterectomy of carotid artery stenosis.

## Methods

### Study Population

Study subjects were recruited from consecutive patients submitted to our stroke unit or attending our outpatient clinic (from September 2005 to August 2008). In total 191 patients were enrolled after written informed consent was obtained. Inclusion criteria were: (1) moderate to high-grade internal carotid artery (ICA) stenosis, diagnosed by Doppler and duplex sonography. The severity of carotid stenosis was evaluated by measuring the peak systolic velocity (PSV) with angle correction at the narrowest point of the stenosis. The degree of stenosis was classified as mild (<199 cm/s), moderate (200-299 cm/s) or severe (≥300 cm/s, or a decrease of PSV combined with distinct duplex sonographic signs of filiform stenosis)[24]. (2) Patients did not present with contraindications for MRI (e.g., pacemakers, metal implants, claustrophobia).

Symptomatic stenosis was defined as a recent (within 3 days prior to enrollment) neurological deficit caused by the index carotid artery.

In all cases the stenosis was caused by atherosclerosis. We used the following diagnostic protocol to determine the stroke etiology and to exclude other possible causes for the qualifying stroke than a symptomatic carotid artery stenosis: In each patient we performed electrocardiography to exclude atrial fibrillation, echocardiography to exclude intracardial thrombus, Doppler sonography of the extra- and intracranial vessels as well as a color-coded duplex ultrasound of the extracranial arteries to exclude carotid artery dissection.

All patients were examined one day before and one day after the endarterectomy by a neurologist who did not have any information regarding the MRI findings. According to the definition of new postprocedural neurological deficits used in large multicenter trials (SPACE[25]; NASCET[26]; ECST[27]), a new postprocedural neurological deficit was diagnosed when lasting longer than 24 hours. Diffusion-weighted imaging (DWI) was performed on the day before as well as on the day after the endarterectomy.

The study protocol was approved by the local ethics committee. Independent data safety monitoring was provided by the Institutional Review Board. The methods used in the study were in accordance with the ethical standards laid down in the 1964 Declaration of Helsinki.

### Risk Factor Evaluation

The clinical examination included physical status, blood pressure measurement, blood tests, 12-lead electrocardiography (ECG) and ultrasound examination of the carotid arteries. DM 2 was defined as a fasting glucose level > 7,0 mmol/l (126 mg/dl), glucose level at any time >11,1 mmol/l (200 mg/dl), use of hypoglycemic agents, or a history of physician-diagnosed DM. Hypertension was defined as systolic blood pressure >140 mmHg or diastolic blood pressure >90 mmHg in the supine position, or use of antihypertensive medication. Hyperlipidemia was defined as a fasting cholesterol value >6,2 mmol/l (240 mg/dl), low-density lipoprotein (LDL) cholesterol >4,9 mmol/l (190 mg/dl), LDL/high-density lipoprotein (HDL) ratio >4.0, or a history of physician-diagnosed increased cholesterol and the use of lipid-lowering medication. Ischemic heart disease was defined as a history of myocardial infarction, angina pectoris, or coronary artery bypass or pathognomonic ECG.

### Endarterectomy

The decision for performing carotid endarterectomy (CEA) was reached in a mulitidisciplinary conference of neurologists, radiologists and vascular surgeons. The involved physicians were unaware of the MRI plaque imaging findings.

The technique of CEA applied in our institution has been reported in detail previously[28-30]. All CEA procedures were performed as classical CEA by two experienced surgeons. The surgeons were blinded to the results of the MRI plaque imaging. All patients received a daily dose of 100 mg aspirin at least 1 day before endarterectomy. The procedure was performed under normotensive, normocapnic general anesthesia. At the beginning of the operation, the patients were anticoagulated with 5000 IU heparin that was subsequently antagonized completely with protamine. After CEA, ASS was continued at a dose of 100 mg per day not time-limited.

### Magnetic Resonance Imaging

#### MRI Plaque Imaging

All patients were imaged with a 1.5-T scanner (Magnetom Symphony Quantum Gradient; Siemens Medical System; Germany) with bilateral phased-array surface coils (PACC-SS15; Machnet B.V., Netherlands). According to our previously published protocol, four contrast-weighted images were obtained as follows:[16] 3-dimensional time-of-flight MR-angiography (3D TOF), T1-weighted (T1w), T2-weighted (T2w), and proton-density-weighted (PDW) studies of both carotid arteries. The MRI scan was centered on the carotid bifurcation on the side of the stenosis to assure proper matching between the contrast-weighted imaging series of each patient. In case of bilateral stenosis, the MRI scan was centered on the side of the more advanced carotid stenosis. The imaging sequences were as follows: 3D TOF: field of view (FOV) 200 mm/75.0%; repetition time (TR) 43 ms; time to echo (TE) 7.15 ms, number of excitations (NEX) 2. T1w: FOV 160 mm/100%; TR 700 ms; TE 14 ms; NEX 2. T2w: FOV 160 mm/100%; TR 700 ms; TE 100 ms; NEX 2. PDW: FOV 160 mm/100%; TR 700 ms; TE 10 ms; NEX 2. Slice thickness was 1 mm for the 3D TOF and 2 mm for the T1w-, T2w- and PDW-images. The longitudinal coverage of each carotid artery was 72 mm (72 slices) for the 3D TOF and 24 mm (12 slices) for T1w, T2w and PDW images.

The patients were positioned on a vacuum pillow to avoid head-neck region movement during the MRI scan to ensure proper alignment between the images acquired in the four contrast-weighted imaging sequences of each patient.

Before evaluation of the MRI scans, an image-quality rating (4-point scale, 1 = best; 4 = worst) was assigned to all MR images for each contrast-weighted image. Image quality = 4 in one of the contrast weightings led to exclusion of the evaluation procedure. For each patient, a data set of 108 contrast-weighted MR images (72 slices for the 3D TOF and 12 slices for T1w, T2w and PDW) of the carotid arteries was obtained. The images were evaluated by one experienced reviewer. The reviewer was blinded to the patient's clinical history and to the histological findings at the time of image analysis. To determine the lesion type in accordance with the modified AHA criteria,[4] the carotid atherosclerotic plaque in the 108 images of each patient was identified and ascribed to one of the six classification types according to the following modified AHA criteria:[4] Type I-II shows near-normal wall thickness without calcification. Type III represents diffuse intimal thickening or small eccentric plaque without calcification. Type IV-V is characterized by a lipid or necrotic core surrounded by fibrous tissue with possible calcification. Type VI shows a complex plaque with possible surface defect, hemorrhage, or thrombus. Type VII represents a calcified lesion. Type VIII is characterized by a fibrotic plaque without a lipid core and with possible small calcifications[4].

### DWI Studies

DWI studies were performed on a 1.5 T whole-body imaging system with a head coil (Magnetom Symphony Quantum gradient). Whole-brain DWI was carried out with an isotropic echo planar sequence. Sagittal, coronal, and transversal studies were obtained, each of them with *b *values of 0, 500, and 1000 s/mm^2^, TR 4006 ms, TE 83 ms, quantum gradient 30 mT/m, slew rate 125 mT/m/ms, rising time 240 μs, slice thickness 4-6 mm, gap 1.5 mm, 128 × 128 matrix and 220 × 220 mm field of view. To minimize the effects of diffusion anisotropy, the diffusion-weighted data were automatically processed by the scanner's software (Numaris^© ^3.5). ADC maps were also automatically processed by the scanner's software. MRI was conducted with special consideration of number and location of lesions. All images were analyzed by one experienced neuroradiologist who was blinded to clinical details including the kind of intervention. An acute ischemic lesion in DWI was diagnosed only if an increased signal intensity was visible on at least two planes, if a corresponding decreased signal intensity was detected in the apparent diffusion coefficient (ADC) image. *Postprocedural DWI lesion *was defined as new, acute ischemic lesion that occurred in the MR-imaging after endarterectomy and that was not in the MR-imaging performed before endarterectomy.

### Histology

Seventy-two patients (40%) underwent carotid endarterectomy and specimens from 36 patients could be obtained for histological work-up. The specimens were fixed in formalin, decalcified, and embedded in paraffin. The samples were sectioned (slice thickness 10 μm) every 1 mm throughout the length of the specimen and stained (hematoxylin-eosin and Mallory's trichrome). The histological sections were independently reviewed and categorized using AHA criteria[31,32]. The reviewer was blinded to the clinical history of the patients and to the results of the MRI evaluation.

### Correlation between MRI and Histology

The region of interest (carotid plaque) was defined in the MR images. The MRI images and histological slices were matched using the distance from the common carotid artery bifurcation and using morphological features as landmarks, such as lumen size and the presence of calcification. Histology was considered the gold standard.

### Statistical Analysis

To compare the frequency of the occurrence of postinterventional DWI lesions with the presence of a symptomatic stenosis and MRI-defined lesion types in diabetic an non-diabetic patients, chi-square tests were used. To assess the association of DM, symptomatic stenosis and cardiovascular risk factors with the presence of high risk lesion types we performed logistic regression analysis. We present odds ratios (OR), adjusted OR and 95% confidence intervals (CI). As exploratory subgroup analyses we also assessed the association of DM adjusted for cardiovascular risk factors with postprocedural lesions in patients that had undergone CEA.

To quantify the degree of concordance between the histological and MRI data, we calculated Cohen's kappa coefficient. A kappa value > 0.75 was considered to indicate good concordance; a kappa value between 0.40 and 0.75 was considered to indicate moderate concordance.

Data were analyzed using SPSS version 16.0 software (SPSS, Chicago, IL, USA). All tests were two-tailed and *P*-values < 0.05 were considered statistically significant.

## Results

One hundred and ninety-one patients were enrolled. Eleven patients (5.8%) had to be excluded because of inadequate MR- image quality (image quality = 4). The following results are based on the remaining 180 patients (94.2%). DM 2 was diagnosed in n = 51 patients (28.3%). Forty-nine patients (27.2%) presented with a recent symptomatic stenosis; 131 patients (72.8%) were asymptomatic. In diabetic patients n = 20 patients (39.2%) presented with a symptomatic stenosis, in nondiabetic patients n = 29 (22.5%). Seventy-two patients (40%) (of whom 27 [37,5%] were symptomatic) underwent CEA. Moderate stenosis (Vmax 200-300 cm/s) was diagnosed in 81 (45%) patients; 99 (55%) patients presented with a high-grade (Vmax ≥ 300 cm/s) carotid stenosis.

Patient demographics and baseline data are summarized in Table 1.

**Table 1:** Baseline data of patients.

	DM 2	No DM 2	*P*
N	51 (28.3)	129 (71.7)	
Symptomatic, n (%)	20 (39.2)	29 (22.5)	0.02
Age, range, mean (years)	51-86 (71.2)	49-87 (71.2)	0.67
Sex, male, n (%)	30 (58.8)	79 (61.2)	0.11
Hypertension, men (%)	43 (84.3)	110 (85.3)	0.87
Atrial Fibrillation, n (%)	2 (3.9)	8 (6.2)	0.55
Current or former smoker, n (%)	28 (54.9)	62 (48.1)	0.41
Hypercholeserolemia, n (%)	31 (60.8)	82 (63.6)	0.73
Coronary heart disease, n (%)	9 (17.6)	39 (30.2)	0.09
Moderate stenosis, n (%)	22 (43.1)	59 (45.7)	0.75
Severe stenosis, n (%)	29 (56.9)	70 (54.3)	0.72

DM 2: Type 2 Diabetes mellitus; N: number.; *P*: *P*-value.

### MRI Lesion Types

**Figure 1:**
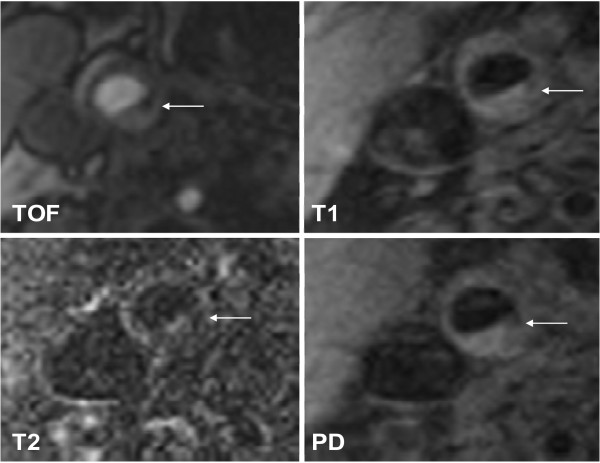
**Example of lesion type IV-V in the right internal carotid artery**. The lipid-rich necrotic core shows low-signal intensity (SI) on both T1w and TOF images, but low- to iso-SI on PDW and T2w images. Original magnification × 25.

**Figure 2:**
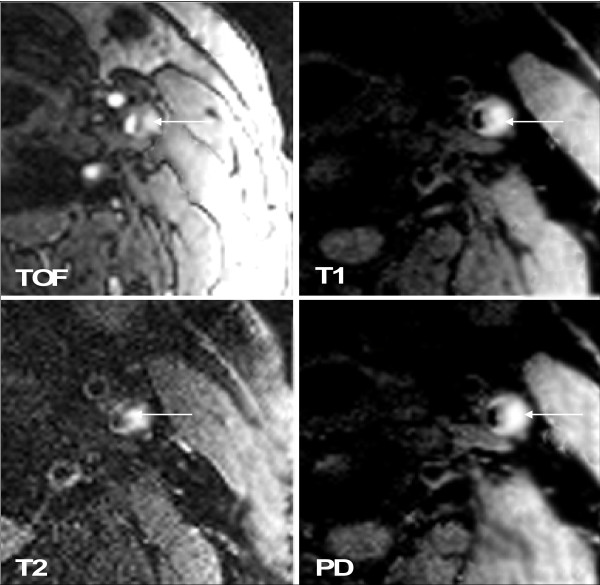
**Example of lesion type VI in the left internal carotid artery**. Intraplaque haemorrhage (←) shows high-SI on T1w, TOF, PDW, and T2w images. Original magnification × 25.

In patients with DM 2, lesion type IV-V (Figure 1) was found most commonly (n = 20; 39.2%), followed by lesion type VII (n = 13; 25.5%), lesion type VI (n = 9; 17.6%) (Figure 2), lesion type VIII (n = 5; 9.8%), and lesion type III (n = 4; 7.8%).

In nondiabetic patients the prevalence of lesion types was as follows: lesion type VII was mainly found (n = 56; 43.4%), followed by lesion type IV-V (n = 28; 21.7%), lesion type VIII (n = 20; 15.5%), lesion type III (n = 15; 11.6%), and lesion type VI (n = 10; 7.8%).

**Figure 3:**
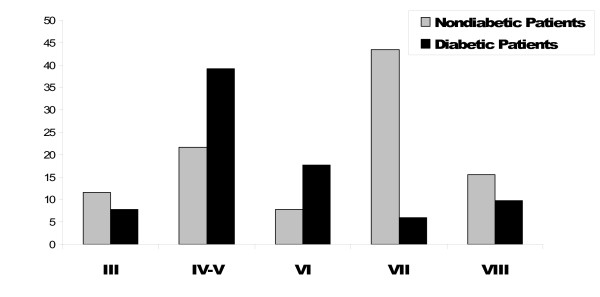
**Distribution of MRI-defined lesion types in patients presenting with DM 2 compared with nondiabetic patients**. The high-risk lesion types IV-V and VI were more prevalent in diabetic patients compared with nondiabetic ones (n = 20 [39.2%] vs. n = 28 [21.7%] and n = 9 [17.6%] vs. n = 10 [7.8%]; *P *= 0.002).

The high-risk lesion types IV-V and VI were more prevalent in diabetic patients compared with nondiabetic patients (n = 29 [56.8%] vs. n = 38 [29.5%]; *P *= 0.002). The distribution of MRI-defined lesion types in diabetic patients compared with nondiabetic patients is shown in Figure 3.

Multiple logistic regression analysis including DM 2, symptomatic stenosis, degreee of stenosis, cholesterol level, hypertension, smoking status, coronary heart disease, and atrial fibrillation as influencing variables revealed association between DM 2 and MRI-defined high-risk lesion types (OR 2.59; 95% CI [1.15-5.81]) and between symptomatic stenosis and MRI-defined high-risk lesion types (OR 13.38; 95%CI [5.64-31.78]). None of the other factors revealed an association in the regression analysis. These data are summarized in Table 2.

**Table 2:** Association between risk factors and the presence of MRI-defined high-risk lesion types in our population of patients presenting with carotid artery stenosis (n = 180).

Variable	Odds Ratio	95% confidence Interval		*P*-value
		**Lower**	**Upper**	

Symptomatic Stenosis	13,35	5.19	34.29	<0.001
Degree of Stenosis	0,84	0.43	1.99	0.843
Diabetes Type II	2,58	1.14	5.84	0.023
Elevated Cholesterol	1,11	0.50	2.50	0.793
Coronary Heart Disease	1,11	0.47	2.60	0.816
Atrial Fibrillation	2,41	0.45	12.76	0.303
Hypertension	0,61	0.21	1.74	0.353
Current or Former Smoker	1,06	0.51	2.22	0.873
Preprocedural DWI-Lesion	1,01	0.36	2.86	0.989

### Histology

Histology data were obtained for 36 patients. The concordance between the histological data and the MRI classification was 91.7% (33/36) and showed a Cohen's kappa value of 0.81 with a 95% CI of 0.98-1.15.

**Figure 4:**
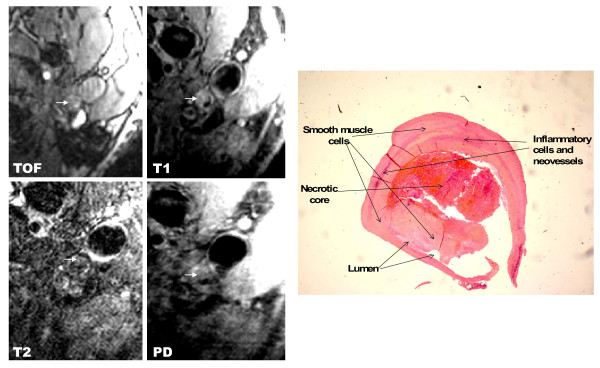
**The image on the left shows an MRI-example of lesion type IV-V in the left internal carotid artery**. The lipid-rich necrotic core shows low- to iso-signal intensity. Original magnification × 25. The image on the right shows the corresponding histological example (lesion type V) in the left internal carotid artery. Hematoxylin-Eosin (HE) Staining. Original magnification × 10.

Figure 4 shows an example of an MRI-defined lesion type IV-V and the corresponding histological image shows an example of lesion type V.

### DWI Lesions

In 25 patients (34.7%) DWI lesions were found before CEA. Twelve of these patients (16.7%) were diabetics.

Seventy-two patients (40%) underwent CEA. Twenty-five (34.7%) of the patients who underwent endarterectomy presented with DM 2.

After endarterectomy postprocedural DWI lesions were found in 25 patients (34.7%). In n = 17 diabetic patients (23.6%) and in n = 6 nondiabetic patients (8.3%) postprocedural DWI lesions occurred.

The logistic regression analysis adjusted for MRI-defined high-risk lesion types, symptomatic stenosis, degree of stenosis and cardiovascular risk factors confirmed this association of DM 2 and postprocedural DWI lesions (OR 5.12; 95% CI [1.01-25.8]).

High-risk MRI lesion types were found in 38 (52.8%) out of the 72 patients who underwent endarterectomy. Diabetic patients with high-risk lesion types developed postprocedural DWI lesions more often than nondiabetic patients with high-risk lesion types (n = 17 [44.7%] vs. n = 6 [15.8%]; *P *= 0.037).

### Neurological Outcome

Considering the definition of new postprocedural neurological deficits used in large multicenter trials (SPACE[25]; NASCET[26]; ECST[27]), postprocedural neurological deficits lasting longer than 24 hours ocurred in n = 4 (5.56%) patients.

All four patients (5.6%) with new neurological symptoms were diabetics.

## Discussion

Our results showed that in a cohort of patients presenting with carotid artery stenosis, patients with type 2 diabetes in particular were at higher risk than nondiabetic patients of presenting vulnerable, carotid plaques: the high-risk lesion types IV-V and VI were clearly overrepresented in diabetic patients compared with nondiabetics. Regression analysis revealed DM 2 to represent an independent risk factor for the presence of high-risk MRI-defined lesion types, irrespective of other cerebrovascular risk factors and irrespective of the degree of stenosis. Our results suggest that DM 2 might play an important role regarding plaque appearance and stability. Since we found in diabetic patients the high-risk MRI-defined lesion types to be predominant, our results suggest that patients with DM 2 might have atherosclerotic patterns different from those of nondiabetic ones with regard to plaque stability. MRI-based plaque imaging represents a new technique to visualize such high-risk plaque features noninvasively and offers therefore a new possibility for risk stratification in diabetic patients.

The hypothesis that atherosclerosis due to DM 2 is different from the pattern of atherosclerosis caused by other risk factors has been suggested by previous literature: Moreno and colleagues[33] found a larger content of high-risk plaque features such as a lipid core in coronary specimens of diabetics than in nondiabetics. Henry et al. found a specific pattern of remodeling in ultrasound examinations, especially in diabetic patients[17]. A possible explanation for the differences in plaque morphology between diabetic patients and nondiabetic patients was shown by the study of Di Mario et al.,[34] since they found an association between hyperglycemia and dysregulation of vascular remodeling. Furthermore, specific features of diabetic atherosclerosis have been identified, such as vasa vasorum neovascularisation leading to intraplaque hemorrhage, and lipid-core expansion leading to high-risk plaque lesions[18]. However, in none of the above studies applied MRI-based plaque imaging for classifying carotid plaque features. We particularly focused on the relation between DM 2 and MRI-based plaque imaging because the latter might offer a new possibility for noninvasive detection of vulnerable plaques in diabetic patients.

Wassermann et al. analyzed the association between cardiovascular risk factors and the presence of a lipid core detected by MRI-based plaque imaging in a cohort of patients without history of cardiovascular disease[35]. In contrast to our results, they found plasma cholesterol, but not DM 2, to be related to a MRI-detected lipid core. However, using MRI-plaque imaging they looked only for one plaque feature, the presence of a lipid core, which represents lesion type IV-V according the modified AHA classification. They did not consider that lesion type VI, characterized by intraplaque hemorrhage or surface defect, also represents a high-risk plaque feature that has been shown by Takaya et al. to be associated with future cerebrovascular events [36]. In contrast, we took into account the analysis of all different lesion types I-VIII in our study. To best use the potential of MRI for detection of high-risk plaques, consideration of each lesion type according to the modified AHA classification is recommended. Furthermore, Wassermann et al. investigated only patients without cardiovascular history [35], whereas we analyzed a cohort presenting with moderate to high-grade carotid stenosis. Since association between DM 2 and high-risk plaque features might be more prevalent in advanced atherosclerotic diseases [18], this might be a further reason for the differences in our results.

Large trials investigating the incidence of postprocedural neurological complications after carotid endarterectomy have shown a higher complication rate in symptomatic stenosis[25] than in asymptomatic stenosis[37]. Since symptomatic stenoses provide a higher risk for perioperative cerebral ischemia than asymptomatic ones, this suggests that symptomatic stenoses are characterized by more high-risk plaque features with a higher risk of embolism during endarterectomy. Indeed, histological studies categorizing plaques in accordance to the American Heart Association (AHA) criteria [31,32] have demonstrated that in particular symptomatic stenoses contain such high-risk plaque features like intraplaque hemorrhage[5-7] and thinned fibrous caps with lipid-rich necrotic cores[8-10]. These plaques are endangered to rupture either spontaneously or especially during the procedure of invasive therapy such as endarterectomy. The high-risk MRI-defined lesion types IV-V and VI are characterized by the presence of a thin fibrous cap[8-10] or intraplaque hemorrhage [5-7], so these lesion types are expected to have a higher risk of complications such as cerebral ischemia due to plaque rupture during endarterectomy. We have previously shown that the high-risk MRI-defined lesion types IV-V and VI are indeed overrepresented in symptomatic stenosis[16], thus underlining the hypothesis that the MRI-defined lesion types IV-V and VI represent high risk, rupture-prone plaques associated with a higher incidence of postprocedural neurological deficits.

We found among patients with MRI-defined high-risk lesion types indeed the diabetic patients to develop postprocedural DWI lesions more often than nondiabetic patients, thus providing further evidence for the hypothesis that carotid plaques of diabetic patients might differ from those of nondiabetics. Our findings suggest that carotid plaques in diabetic patients are more likely to rupture during CEA than in other patients.

After adjustment for other risk factors, DM 2 remained an independent predictor for postprocedural DWI lesions. Since diabetic patients with MRI-detected high-risk lesion types seem to be at higher risk for the development of postprocedural ischemia than nondiabetics with high-risk lesions, more intensive monitoring before and during invasive procedures should be applied for these endangered patients and preventive strategies should be adopted.

The association of DM with postprocedural neurological outcome has been discussed controversial so far: Mathur et al.[38] (n = 231 patients) and Qureshi et al.[39] (n = 111 patients) did not observe any relation between DM and periprocedural neurological symptoms. In the European Carotid Surgery Trial (ECST)[27] and the North American Symptomatic Endarterectomy Trial (NASCET) [26], DM was associated with a higher rate of perioperative stroke or death. However, in none of the above studies was DWI analysis performed routinely to find clinically silent lesions that caused no detectable neurological deficit.

Since in our study regression analysis revealed DM 2 to be associated with high risk lesion types and postprocedural DWI lesions independent of the degree of stenosis, our results suggest that DM seems to play a role in the development of high-risk, rupture prone plaques irrespective of the degree of vessel narrowing. Our findings provide further evidence that plaque morphology and composition seems to be more predictive for plaque vulnerability than plaque burden.

A limitation of our study is the low number of histological specimens. Furthemore, we did not use contrast agents for the MRI plaque imaging, so direct visualisation of distinct markers of plaque vulnerability such as macrophage density was not possible.

However, our study represents promising initial data regarding the predictive value of MRI plaque imaging, but larger studies are needed to confirm our results.

## Conclusion

In conclusion, our data suggest that DM 2 irrespective of other cerebrovascular risk factors and irrespective of the degree of stenosis represents a predictor for the presence of vulnerable carotid plaques detected by MRI-based plaque imaging. Carotid plaques of diabetic patients seem to be more in danger of rupture during endarterectomy than carotid plaques of nondiabetic patients. MRI-based plaque imaging seems to represent a promising future method for risk stratification in atherosclerotic patients.

## Competing interests

The authors declare that they have no competing interests.

## Authors' contributions

LE: has made substantial contributions to conception and design, and acquisition of data, and analysis and interpretation of data; has written the manuscript. OP: has made substantial contributions to conception and design and analysis and interpretation of data. HP: has made substantial contributions to conception and design, and acquisition of data, and analysis and interpretation of data. He performed carotid anderterectomy of study patients and carried out the histological analysis. AB: has made substantial contributions to analysis and interpretation of data; has performed the statistical analysis; helped to draft the manuscript. TS: has made substantial contributions to analysis and interpretation of data; has performed the MRI plaque imaging analysis. DS: has made substantial contributions to conception and design and analysis and interpretation of data. RF: has made substantial contributions to conception and design and analysis and interpretation of data. CW: has made substantial contributions to conception and design and analysis and interpretation of data. TL: has made substantial contributions to analysis and interpretation of data; has performed the MRI - analysis. KH: has made substantial contributions to conception and design and analysis and interpretation of data. JP: has made substantial contributions to conception and design and analysis and interpretation of data, has performed histological analysis. SS: has made substantial contributions to conception and design and analysis and interpretation of data. BH: has made substantial contributions to conception and design and analysis and interpretation of data; helped to draft the manuscript. HP: has made substantial contributions to conception and design and analysis and interpretation of data; helped to draft the manuscript.

## Pre-publication history

The pre-publication history for this paper can be accessed here:

http://www.biomedcentral.com/1471-2342/10/27/prepub
